# A role for vaccinia virus protein C16 in reprogramming cellular energy metabolism

**DOI:** 10.1099/vir.0.069591-0

**Published:** 2015-02

**Authors:** Michela Mazzon, Cecilia Castro, Lee D. Roberts, Julian L. Griffin, Geoffrey L. Smith

**Affiliations:** 1 Department of Pathology, Tennis Court Road, University of Cambridge, Cambridge CB2 1QP, UK; 2 Department of Biochemistry and Cambridge Systems Biology Centre, Tennis Court Road, University of Cambridge, Cambridge CB2 1GA, UK; 3 Medical Research Council Human Nutrition Research, Elsie Widdowson Laboratory, Fulborn Road, Cambridge CB1 9NL, UK

## Abstract

Vaccinia virus (VACV) is a large DNA virus that replicates in the cytoplasm and encodes about 200 proteins of which approximately 50 % may be non-essential for viral replication. These proteins enable VACV to suppress transcription and translation of cellular genes, to inhibit the innate immune response, to exploit microtubule- and actin-based transport for virus entry and spread, and to subvert cellular metabolism for the benefit of the virus. VACV strain WR protein C16 induces stabilization of the hypoxia-inducible transcription factor (HIF)-1α by binding to the cellular oxygen sensor prolylhydroxylase domain-containing protein (PHD)2. Stabilization of HIF-1α is induced by several virus groups, but the purpose and consequences are unclear. Here, ^1^H-NMR spectroscopy and liquid chromatography-mass spectrometry are used to investigate the metabolic alterations during VACV infection in HeLa and 2FTGH cells. The role of C16 in such alterations was examined by comparing infection to WT VACV (strain WR) and a derivative virus lacking gene *C16L* (vΔC16). Compared with uninfected cells, VACV infection caused increased nucleotide and glutamine metabolism. In addition, there were increased concentrations of glutamine derivatives in cells infected with WT VACV compared with vΔC16. This indicates that C16 contributes to enhanced glutamine metabolism and this may help preserve tricarboxylic acid cycle activity. These data show that VACV infection reprogrammes cellular energy metabolism towards increased synthesis of the metabolic precursors utilized during viral replication, and that C16 contributes to this anabolic reprogramming of the cell, probably via the stabilization of HIF-1α.

## Introduction

*Vaccinia virus* (VACV) is the prototypic member of the genus *Orthopoxvirus* of the family *Poxviridae*. VACV is also the live vaccine used to eradicate smallpox (Fenner *et al.*, 1988). Long after smallpox eradication, VACV is still studied intensively due to its role in vaccine development and because VACV is an excellent model for studying virus–host interactions, such as immune evasion (Smith *et al.*, 2013).

VACV is a large, dsDNA virus encoding about 200 genes (Goebel *et al.*, 1990) that replicates in the cytoplasm (Moss, 2007) and reprogrammes cell biochemistry to favour viral replication. For example, VACV encodes a growth factor (Twardzik *et al.*, 1985) that stimulates the metabolism of resting cells (Buller *et al.*, 1988a, b) and several enzymes involved in nucleotide metabolism, such as ribonucleotide reductase (Slabaugh *et al.*, 1988; Tengelsen *et al.*, 1988), thymidine kinase (Hruby & Ball, 1982; Weir *et al.*, 1982), thymidylate kinase (Hughes *et al.*, 1991; Smith *et al.*, 1989b), dUTPase (Broyles, 1993; McGeoch, 1990) and uracil DNA glycosylase (De Silva & Moss, 2003, 2008). VACV also encodes three thiol oxidoreductases enabling protein disulphide bond formation (Senkevich *et al.*, 2002; White *et al.*, 2000), a steroid biosynthetic enzyme to suppress inflammation (Moore & Smith, 1992; Reading *et al.*, 2003), protein kinases (Banham & Smith, 1992; Lin & Broyles, 1994; Lin *et al.*, 1992; Rempel & Traktman, 1992), a phosphatase (Guan *et al.*, 1991), DNA ligase (Colinas *et al.*, 1990; Kerr & Smith, 1989; Smith *et al.*, 1989a) and topoisomerase (Shuman & Moss, 1987). For a review of enzymes involved in DNA replication, see Moss (2013). Very recently, two studies reported increased glutamine utilization (Fontaine *et al.*, 2014) and *de novo* fatty acid biosynthesis (Greseth & Traktman, 2014) during VACV infection.

VACV protein C16 is an intracellular virulence factor (Fahy *et al.*, 2008) that binds the cellular oxygen sensor prolylhydroxylase domain-containing protein (PHD)2, thereby preventing hydroxylation and consequent degradation of hypoxia-inducible transcription factor (HIF)-1α during normoxia (Mazzon *et al.*, 2013). HIF-1α is a transcriptional regulator of oxygen homeostasis. During hypoxia, HIF-1α is stabilized and translocates into the nucleus where it induces transcription of numerous genes. These include genes encoding proteins that promote oxygen uptake, by inducing angiogenesis and erythropoiesis, and decreasing oxygen consumption, by switching metabolism from oxidative phosphorylation, which generates ATP and consumes oxygen in the mitochondrion, to glycolysis (Semenza, 2012). Interestingly, HIF-1α stabilization and the consequent metabolic shift occur during normoxia in several physiological and pathological conditions, particularly in rapidly proliferating cells, or upon infection with several viral and non-viral pathogens (Carroll *et al.*, 2006; Tang *et al.*, 2007; Werth *et al.*, 2010). These observations suggest there are other advantages to pathogens from HIF-1α stabilization beyond regulation of oxygen homeostasis, as reviewed by Chen & Russo (2012). It is possible that HIF-1α stabilization might play a role in VACV reprogramming of cellular metabolism.

The emerging field of viral metabolomics is helping explain how viruses reprogramme cell metabolism to assist viral replication (Diamond *et al.*, 2010; Heaton *et al.*, 2010; Vastag *et al.*, 2011). Here, a combination of proton nuclear magnetic resonance (^1^H-NMR) spectroscopy and liquid chromatography-mass spectrometry (LC-MS) is used to investigate the metabolic alterations induced by VACV infection in two cell types, focusing on alterations in energy metabolism that might be influenced by stabilization of HIF-1α. An increase in nucleotides and intermediates of glucose and glutamine metabolism was observed. Interestingly, no increase in lactate production or decrease in tricarboxylic acid (TCA) cycle activity was seen, as might have been expected following stabilization of HIF-1α. Moreover, the increase in glutamine metabolism following infection with WT VACV was much diminished following infection with a virus-lacking gene *C16L* (vΔC16), implicating protein C16 in reprogramming cellular energy metabolism.

## Results

### Metabolic profile of VACV-infected HeLa cells

HIF-1α stabilization induces alterations in cellular energy metabolism (Semenza, 2012). Given that VACV protein C16 induces rapid stabilization of HIF-1α and upregulation of hypoxia-inducible genes (Mazzon *et al.*, 2013), we hypothesized that protein C16 might reprogramme cellular metabolism.

The metabolic alterations following infection by VACV strain WR or a derivative virus lacking C16 (vΔC16) were characterized in HeLa cells, a human carcinoma cell line used widely for studying virus infections. Cells were grown to confluency to reduce cell proliferation and induce some degree of synchrony, while minimum essential medium (MEM) with low serum (1 %) and low glucose concentrations (1 g l^−1^) was used to reduce cellular metabolic activity. Cells were mock-infected or infected with VACV or vΔC16, and intracellular aqueous metabolites were extracted and analysed by ^1^H-NMR spectroscopy, a technique suitable for studying central energy metabolism. Partial least squares-discriminant analysis (PLS-DA) was used to analyse the datasets. PLS-DA allows analysis and group discrimination between a large number of variables (here, the concentration of metabolites), and subsequent identification of the variables contributing to the discrimination. Results are expressed in terms of ‘score plots’, which highlight patterns and clustering among the samples (each point in the plots represents one sample and there are five replicates per condition), and ‘loadings’, or ‘assignment lists’, which indicate the importance of each original variable (metabolite). Metabolites listed on the left-hand side are more abundant in samples displayed in the negative area of the t(1) axis, while metabolites listed on the right-hand side are more abundant in samples in the positive area. It is worth mentioning that in NMR each metabolite is associated with more than one signal, and each signal is usually large enough to be present in two or more adjacent buckets (the discrete partitions into which each NMR spectrum is divided for integration). This leads to multiple loading values for each metabolite, as seen in the ‘redundancy’ of assignment lists next to the score plots and in the corresponding supplementary tables. Data are presented following standard presentation criteria as described by van der Werf *et al.* (2007), Martin *et al.* (2007) and Sumner *et al.* (2007).

**Fig. 1.**
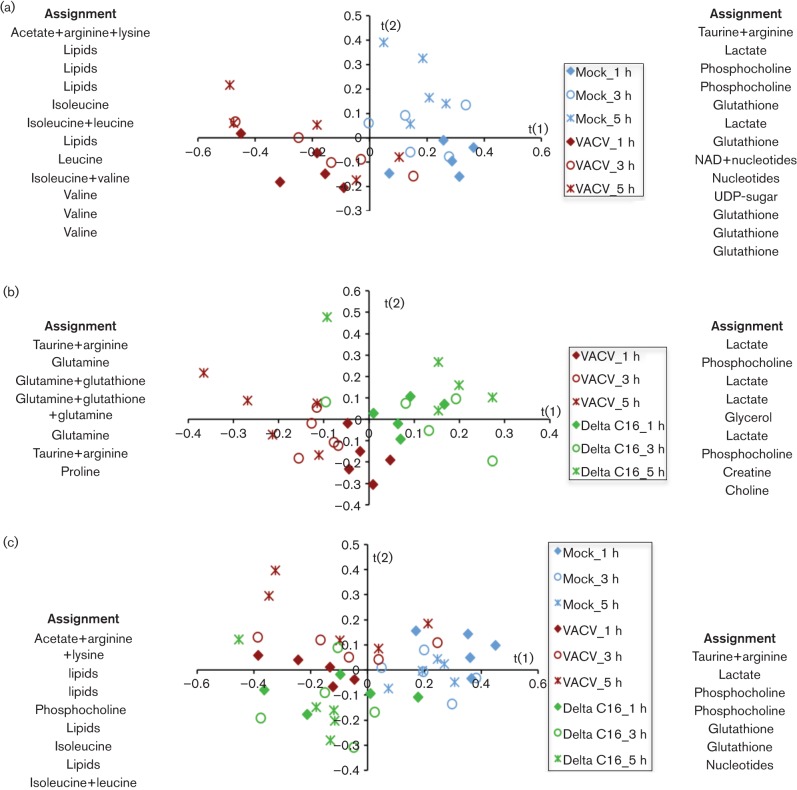
Metabolic profile of HeLa cells. Five T175 flasks of confluent HeLa cells were either infected with VACV or vΔC16 or mock-infected and at the indicated times pi, cells from each flask were analysed separately for ^1^H-NMR spectroscopy. (a), (b) and (c) show score plots for the PLS-DA models obtained using data from cells infected with either VACV (red symbols), vΔC16 (green symbols) or mock-infected (blue symbols), at 1, 3 and 5 h pi (diamonds, circles and asterisks, respectively). Each point in the graph represents one sample. Assignment lists at either side of each score plot show the metabolites responsible for the separation in the profiles obtained: metabolites listed on the left are more abundant in samples displayed in the negative area of the t(1) axis, while metabolites listed on the right are more abundant in samples in the positive area. Complete loading plots are shown in Tables S2, 3 and 4. The model parameters are: R^2^(X) = 67 %, R^2^(Y) = 84.2 %, Q^2^ = 55 % (a); R^2^(X) = 70.7 %, R^2^(Y) = 99 %, Q^2^ = 87.4 % (b); and R^2^(X) = 65.6 %, R^2^(Y) = 98.4 %, Q^2^ = 73.5 % (c).

In HeLa cells, 27 metabolites were identified (Table S1, available in the online Supplementary Material). Application of PLS-DA to the datasets separated mock-infected from WT VACV (VACV)-infected HeLa cells (Fig. 1a). The assignment lists at each side of the graphs illustrate global metabolic changes in the datasets, with metabolites listed on the left being more important in defining infected samples and indicating a higher abundance of lipids following VACV infection (Fig. 1a) or vΔC16 (Fig. 1a, c, Tables S2 and S4). Pairwise comparison also distinguished infection with WT VACV from vΔC16, with higher levels of glutamate, glutamine, glutathione and taurine in cells infected by WT VACV (Fig. 1b, Table S3). The differences between these viruses increased with time, with maximal separation at 5 h post-infection (pi) (Fig. 1b, c), consistent with the progressive onset of different metabolic programmes in the presence or absence of C16, but were not as large as the differences between infected and mock-infected samples (Fig. 1c).

**Fig. 2.**
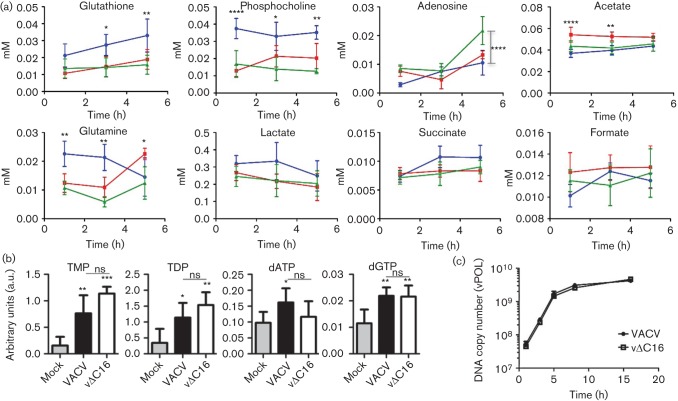
Comparison of metabolite concentrations in infected HeLa cells. (a) Data obtained as in Fig. 1 are presented graphically to show the concentration of assigned metabolites from 1–5 h pi in VACV-infected (red line), vΔC16-infected (green line) or mock-infected HeLa cells (blue line). Concentrations are in mM. Data were analysed using a two-way ANOVA with Bonferroni’s correction. Apart from where indicated differently, statistical significance is shown for comparisons between mock and VACV-infected samples. Data represent mean±SEM and experiments were performed using five replicates per sample. *P<0.05; **P<0.01; ***P<0.001; ****P<0.0001. (b) Nucleotide concentrations at 5 h pi. HeLa cells (T175 flasks *n* = 5) were infected with VACV or vΔC16, or were mock-infected and at 5 h pi samples were processed for LC-MS. The *y*-axis shows integrated peak areas. Data were analysed using a one-way ANOVA, *P<0.05; **P<0.01; ***P<0.001. Differences between VACV and vΔC16 were non-significant (ns). (c) Viral DNA synthesis in HeLa cells infected with VACV or vΔC16 at 10 p.f.u. per cell. DNA was harvested and quantified at the indicated times pi. Amplification of viral DNA polymerase DNA was quantified by PCR. One representative of two experiments is shown.

When measuring concentrations of individual metabolites with time, lower concentrations of several metabolites, particularly glutamine and phosphocholine (at each time point) and glutathione (at 1 and 3 h pi), were measured with each virus compared with mock-infected samples (Fig. 2a). The concentration of adenosine increased at 5 h pi for both viruses, reaching statistical significance for vΔC16 compared with mock (*P*<0.001), while levels of glutamine, initially higher in mock-infected cells, were higher in cells infected with VACV by 5 h pi (*P*<0.05) compared with mock. Surprisingly, in the cells (Fig. 2a) and culture medium (data not shown), an increase in lactate was not seen after infection with either virus compared with mock. For WT VACV, this was unexpected due to the stabilization of HIF-1α that promotes glycolysis. Furthermore, there was no decrease in TCA cycle activity following infection with either virus, as indicated by unchanged levels of TCA cycle intermediates succinate and formate. Changes here might also have been expected following HIF-1α stabilization.

Even though PLS-DA was able to separate VACV- from vΔC16-infected cells clearly (Fig. 1b), only minor differences were observed in the concentrations of individual metabolites in HeLa cells infected with these viruses at these times (Fig. 2a). This indicates that the difference between the two groups is associated with global changes in the relative abundance of metabolites within each group, rather than with individual concentrations. For instance, although assignment lists (Fig. 1b) suggest reduced accumulation of lactate in cells expressing C16 (i.e. where HIF-1α is stabilized) this is not statistically significant (Fig. 2a).

For large DNA viruses, DNA replication requires high levels of nucleotides and it was estimated that VACV synthesizes 10 000 copies of its 200 kbp genome within a few hours in the cytoplasm (Joklik & Becker, 1964). Some herpes viruses induce increased nucleotide biosynthesis after infection (Vastag *et al.*, 2011). An increase in adenosine was seen in HeLa cells 5 h pi with vΔC16 (Fig. 2a) and, therefore, the nucleotide profile of infected HeLa cells was analysed more fully by LC-MS and the metabolites identified are shown in Table S5. As observed by ^1^H-NMR spectroscopy, several metabolites were either unchanged or reduced upon infection (not shown). However, concentrations of TMP, TDP, dGTP and dATP were significantly higher following infection (Fig. 2b). Increased concentrations of TMP and TDP, observed upon infection with either virus, are consistent with the early expression of the VACV-encoded thymidine kinase (Hruby & Ball, 1982; Weir *et al.*, 1982) and thymidylate kinase (Hughes *et al.*, 1991; Smith *et al.*, 1989b).

To verify that global metabolic differences observed between the two viruses (Fig. 1b) were not attributable to differences in replication rate, HeLa cells were infected with VACV or vΔC16 and viral DNA was quantified by measuring gene *E9L* abundance (encoding DNA polymerase, vPOL; Jones & Moss, 1984) by PCR. DNA synthesis was similar for each virus, with viral DNA accumulating from shortly after infection and reaching a plateau by 5 h (Fig. 2c). These data are consistent with a previous report showing these viruses replicated equally well *in vitro* (Fahy *et al.*, 2008), and suggest that differences in the metabolic profile of cells infected with these viruses are not caused by different viral DNA replication rates. These data also indicate that any metabolic alteration observed is unlikely to affect viral replication in the cultured cells examined.

### Characterization of VACV infection in 2FTGH cells

**Fig. 3.**
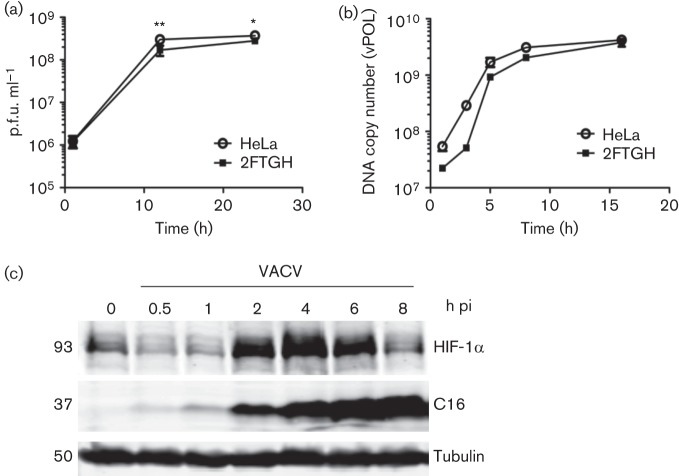
Infection of 2FTGH with VACV. HeLa or 2FTGH cells were infected with VACV at 10 p.f.u. per cell and infectious virions (a) or viral DNA (b) were quantified at the indicated times. (a) Infectious intracellular virions were titrated by plaque assay. (b) Amplification of gene *E9L* DNA was quantified by PCR. Data shown are from one representative experiment of two experiments that gave indistinguishable data. Data were analysed using a two way ANOVA with Bonferroni’s correction. *P<0.05; **P<0.01. (c) Stabilization of HIF-1α in VACV-infected 2FTGH cells. Cells were infected with VACV at 5 p.f.u. per cell and at the time points indicated, cells were lysed and the levels of HIF-1α, VACV protein C16 and α-tubulin were analysed by SDS-PAGE and immunoblotting. Molecular mass markers are indicated on the left in kDa. One representative experiment of two experiments is shown.

In HeLa cells, significant differences between infected and mock-infected cells were observed by 1 h pi and were accompanied by reduction in several metabolites (Fig. 2a), reminiscent of the rapid shut-down of cellular transcription in VACV-infected HeLa cells (Guerra *et al.*, 2003; Moss, 2007). To investigate if VACV infection altered intracellular metabolites in other cells, a human fibrosarcoma cell line, 2FTGH, was utilized. This cell line was selected because we had observed a slower and more gradual progression of VACV infection compared to HeLa cells, and therefore its use increased the opportunity to see metabolic differences at early time points after viral entry. Characterization of viral replication (Fig. 3a) and DNA synthesis (Fig. 3b) showed that each were slightly slower than in HeLa cells, although at 24 h pi the final difference in virus titre was quite small. VACV-induced stabilization of HIF-1α, already described in HeLa cells (Mazzon *et al.*, 2013), was also seen in 2FTGH cells (Fig. 3c), although this occurred slightly more slowly than in HeLa cells, with maximal accumulation between 2 and 6 h pi.

### Metabolic profile of 2FTGH cells after infection

**Fig. 4.**
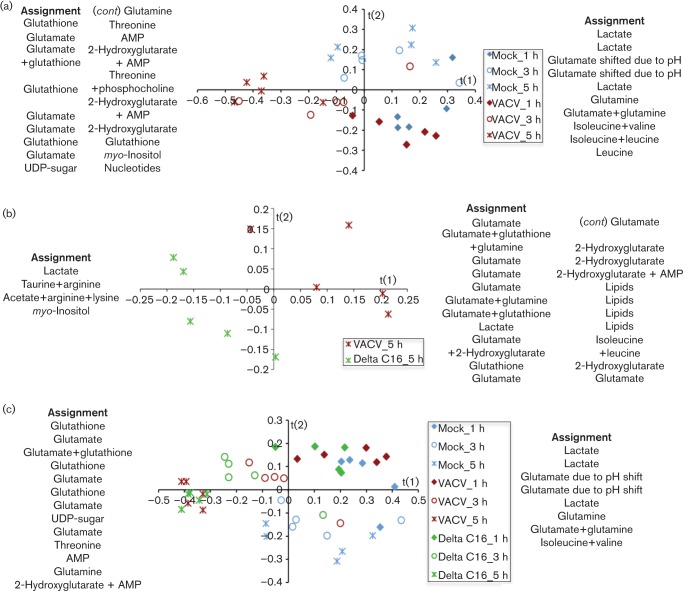
Metabolic profile of infected 2FTGH cells. Five T175 flasks of confluent 2FTGH cells were infected with VACV or vΔC16 or were mock-infected and when indicated cells from each flask were analysed separately for ^1^H-NMR spectroscopy. (a), (b), and (c) show score plots for the PLS-DA models, obtained using data from cells infected with VACV (red symbols), or vΔC16 (green symbols), or mock-infected (blue symbols) at 1, 3 and 5 h pi (diamonds, circles and asterisks, respectively). Each point in the graph represents one sample. Assignment lists show the metabolites responsible for the separation in the profiles obtained: metabolites listed on the left are more abundant in samples displayed in the negative area of the t(1) axis, while metabolites listed on the right are more abundant in samples on the positive area. Complete loading plots are shown in Tables S7, S8 and S9. The model parameters are: R^2^(X) = 78.7 %, R^2^(Y) = 64.5 %, Q^2^ = 30.5 % (a); R^2^(X) = 78.9 %, R^2^(Y) = 90.1 %, Q^2^ = 59.7 % (b); R^2^(X) = 48.1 %, R^2^(Y) = 88.5 %, Q^2^ = 53.2 % (c).

To analyse virus-induced metabolic alterations, confluent 2FTGH cells were infected with VACV or vΔC16, and metabolites were measured by ^1^H-NMR spectroscopy as for HeLa cells. The 32 metabolites identified are shown in Table S6. Application of PLS-DA to the datasets separated infected from mock-infected 2FTGH cells (Fig. 4a, c), but clear metabolic differences between samples were apparent after 3 h, indicating that metabolic reprogramming is slightly delayed compared to HeLa cells. Global metabolic changes in the datasets are illustrated in the assignment lists at each side of the score plots, which revealed increased concentrations of glutamine, glutamate, glutathione and the onco-metabolite 2-hydroxyglutarate (2-HG) in infected samples compared to mock-infected cells (Fig. 4a, c, Tables S7 and S9).

As in HeLa cells, PLS-DA also separated cells infected with VACV and vΔC16, but only from 5 h pi (Fig. 4b, c). Assignment lists show that higher levels of glutamate, glutamine, and glutathione accumulated in cells infected by VACV than by vΔC16 (Fig. 4b, c, Tables S8 and 9), and this was consistent with observations in HeLa cells (Fig. 1b, c). 2-HG is a by-product of reductive carboxylation of glutamine (Wise *et al.*, 2011) and accumulated in VACV-infected cells, and was one of the most critical metabolites to discriminate between VACV and vΔC16. Higher abundance of glutamine, glutamate, glutathione, and 2-HG in cells infected with VACV indicate a role for C16 in supporting and enhancing reductive carboxylation of glutamine.

**Fig. 5.**
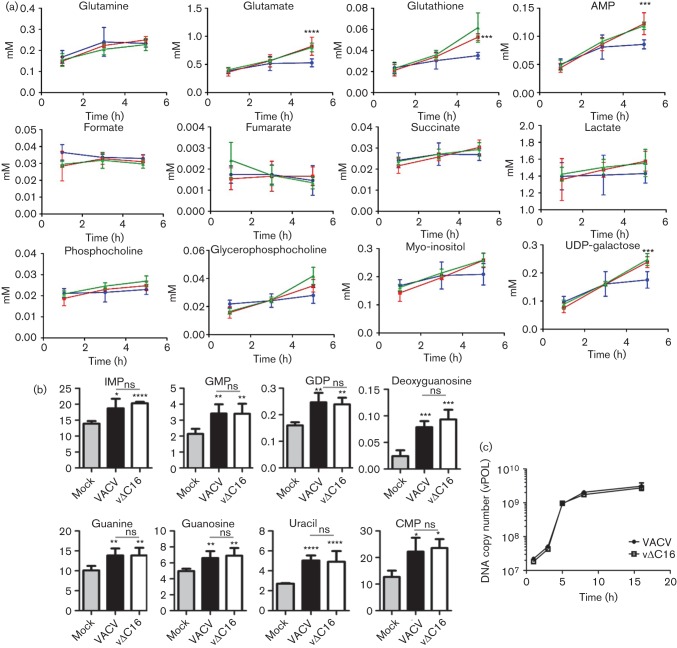
Comparison of 2FTGH cells infected with VACV or vΔC16. (a) Data obtained as in Fig. 4 are presented graphically to show the concentration of assigned metabolites from 1–5 h pi in VACV-infected (red line), vΔC16-infected (green line) or mock-infected 2FTGH cells (blue line). Concentrations are in mM. Statistical analysis was as in Fig. 2a. (b) Nucleotide concentrations at 5 h pi. 2FTGH cells (T175 flasks, *n* = 5) were infected with VACV or vΔC16 or were mock-infected and at 5 h pi samples were processed for LC-MS. The *y*-axis shows integrated peak areas. Statistical analyses are as in Fig. 2b. (c) Viral DNA synthesis in 2FTGH cells infected with VACV or vΔC16 at 10 p.f.u. per cell. DNA was harvested and quantified at the indicated times pi. Gene *E9L* DNA was quantified by PCR. Data shown are from one of two experiments that gave indistinguishable results.

In infected 2FTGH cells several metabolites increased progressively with time compared to mock-infected cells (Fig. 5a) and this differed somewhat from the generic decrease in the metabolic pool observed in HeLa cells. In particular, the levels of glutathione, glutamate, AMP, and two products of glucose metabolism, *myo*-inositol and UDP-galactose, all increased in infected 2FTGH cells, consistent with the PLS-DA. However, consistent with observations in HeLa cells, there was no significant difference between infected and mock-infected 2FTGH cells in concentrations of lactate or TCA cycle intermediates, showing that the stabilization of HIF-1α does not promote aerobic glycolysis or prevent TCA cycle activity in these *in vitro* settings.

As also observed in HeLa cells, whilst the PLS-DA analysis shows profound differences among 2FTGH cells infected with VACV and vΔC16, only minor differences could be observed in many individual metabolites between these two groups. This again suggests larger alterations in the relative abundance of different metabolites within each group, rather than in the concentrations of individual metabolites.

The nucleotide profile of infected 2FTGH cells also was analysed in more detail at 5 h pi by LC-MS and compared to mock-infected cells. As seen by ^1^H-NMR spectroscopy, in 2FTGH cells all nucleotides detected were upregulated during infection, but in particular, significantly higher concentrations of GTP precursors GMP, GDP, guanine, guanosine, IMP and deoxyguanosine were observed. A statistically significant increase in CMP and uracil was also measured, confirming that in both cell lines VACV increases the nucleotide pool of infected cells (Fig. 5b). No differences in the replication rates of VACV and vΔC16 was observed in 2FTGH cells (Fig. 5c), confirming that the differences observed are not dependent on different replication levels of these viruses.

### Metabolic profile of 2FTGH cells at 7 h pi

**Fig. 6.**
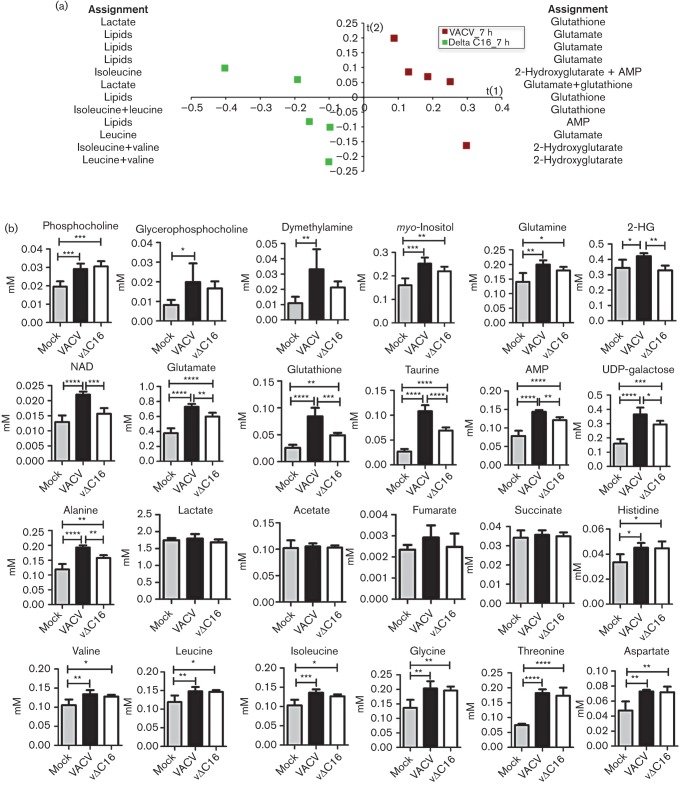
Metabolic profile of 2FTGH cells 7 h pi. Five T175 flasks of confluent 2FTGH cells were infected with VACV or vΔC16 or were mock-infected and at 7 h pi, cells from each flask were analysed separately for ^1^H-NMR spectroscopy. (a) Score plots for the PLS-DA model were obtained using data from cells infected with VACV (red squares) or vΔC16 (green squares). Each point in the graph represents one sample. Assignment lists show the metabolites responsible for the separation in the profiles obtained: metabolites listed on the left are more abundant in samples displayed in the negative area of the t(1) axis, while metabolites listed on the right are more abundant in samples in the positive area. Complete loading plots are shown in Table S10. The model parameters are: R^2^(X) = 88.3 %, R^2^(Y) = 99.2 %, Q^2^ = 92.6 %. (b) Data obtained as in (a) are presented graphically to show the concentration of assigned metabolites at 7 h pi in VACV-infected, vΔC16-infected or mock-infected 2FTGH cells. Concentrations are in mM. Data were analysed using a one-way ANOVA. *P<0.05; **P<0.01; ***P<0.001; ****P<0.0001.

In 2FTGH cells the differences following infection with VACV or vΔC16 increased with time (as in HeLa cells), and this is consistent with the progressive accumulation of different sets of metabolites following infection. In light of this, and because of the slightly slower stabilization of HIF-1α in infected 2FTGH cells compared with HeLa cells, the metabolic differences between 2FTGH cells infected with VACV, vΔC16 or mock-infected were also compared at 7 h pi (Fig. 6a, b, Table S10). The separation between VACV and vΔC16 had greater statistical significance at 7 h (Q^2^ = 92.6 %, Fig. 6a) versus 5 h (Q^2^ = 59.7 %, Fig. 4b). In particular, even though higher levels of intermediates of glutamine metabolism (glutamine, glutamate, 2-HG, glutathione) were observed following infection with either VACV or vΔC16, significantly higher levels of all these metabolites were observed upon infection with VACV than vΔC16, consistent with PLS-DA and loading plots. Interestingly, 2-HG (together with NAD^+^) increased significantly only during infection with WT VACV, indicating higher reductive carboxylation of glutamine in the presence of C16. The TCA intermediates, succinate and fumarate, remained similar between mock-infected and infected cells (either virus), as already observed at all earlier time points in both cell lines, indicating that the activity of the TCA cycle is preserved throughout the infection. Higher levels of most amino acids were also observed upon infection with either VACV or vΔC16 compared with mock-infected cells, but no significant difference was observed between the two viruses, suggesting that the metabolic differences between VACV and vΔC16 are specific to energy metabolism.

Taken together, the data presented show that, despite some cell-specific differences affecting primarily comparison to mock-infected cells, VACV induces a systematic reprogramming of cell metabolism characterized by increased nucleotide synthesis and extensive utilization of glutamine. Possibly, glutamine may be used as the carbon source to support the TCA cycle and the synthesis of new molecules required for viral replication.

The differences between VACV and vΔC16 show that protein C16 contributes to reprogramming of central metabolism by VACV, and in particular to increased utilization of glutamine. Although increased utilization of glutamine is observed during HIF-1α stabilization, the metabolic profile observed differs from a canonical Warburg effect. This suggests either that HIF-1α stabilization has different and more complex metabolic consequences than increasing aerobic glycolysis, particularly in promoting utilization of glutamine, as recently reported by several studies (Metallo *et al.*, 2012; Wise *et al.*, 2011), or that C16 acts on cell metabolism through more than one mechanism.

## Discussion

VACV is a complex DNA virus that continues to provide insight into pathogen–host interactions. VACV infection has a dramatic impact on cells, but the metabolic alterations that accompany the early stages of VACV infection have only recently started to be investigated. Here, the metabolic changes associated with VACV infection of HeLa and 2FTGH cells at early time points were examined, using a combination of ^1^H-NMR spectroscopy and LC-MS, and the consequence of C16-mediated stabilization of HIF-1α on cellular metabolism was also studied.

A comparison of the response of HeLa and 2FTGH cells to infection showed some cell-specific differences and several similarities. The main differences were seen in comparison with mock-infected cells, possibly reflecting a different impact of viral replication of different metabolic backgrounds. However, a remarkably conserved feature was the increase in glutamine and nucleotide metabolism as infection progressed from 1 to 5–7 h, and an even more sustained induction of glutamine metabolism in the presence of C16.

Previously we showed that early after infection, VACV protein C16 induces stabilization of HIF-1α by inhibiting the enzymatic activity of PHD2 (Mazzon *et al.*, 2013). HIF-1α is also stabilized by a wide range of pathogens (Carroll *et al.*, 2006; Darekar *et al.*, 2012; McFarlane *et al.*, 2011; Nakamura *et al.*, 2009; Nasimuzzaman *et al.*, 2007; Werth *et al.*, 2010), and because infections can modulate several signalling pathways, HIF-1α stabilization might contribute to different metabolic outcomes in a context-dependent manner. Whilst HIF-1α induces a shift towards glycolysis in hypoxia (Semenza, 2012), its stabilization in normoxia is known to have advantages beyond oxygen preservation.

Even though stabilization of HIF-1α was seen in both the cell lines studied, evidence of a canonical Warburg effect, i.e. increased lactate production and decreased TCA cycle activity, markers of a metabolic shift towards glycolysis, were not observed. Conversely, the activity of the TCA cycle was preserved and was likely to have been promoted by increased utilization of glutamine. To investigate whether C16 has a role in metabolic changes, possibly through stabilization of HIF-1α, the metabolic profiles of cells infected with WT VACV or a virus lacking C16 (Fahy *et al.*, 2008; Mazzon *et al.*, 2013) were studied and showed that glutathione and intermediates of glutamine reductive carboxylation (glutamine, glutamate and 2-HG) were significantly higher in 2FTGH cells infected by VACV than by vΔC16. Conversely, no difference could be detected in nucleotide or amino acid synthesis or in phosphocholine metabolism, suggesting that these selective differences are specific to pathways involved in central energy metabolism.

Particularly interesting was the accumulation of 2-HG, predominantly in cells infected with VACV. 2-HG is an onco-metabolite that is not normally observed in cells unless isocitrate dehydrogenase is mutated, which has been observed in several tumours. Increased concentration of 2-HG suggests enhanced reverse flux of α-ketoglutarate through isocitrate dehydrogenase, and this phenomenon can occur following increased utilization of glutamine during HIF-1α stabilization (Wise *et al.*, 2011).

Recent reports have shown that an increase in glutamine utilization can be observed upon stabilization of HIF-1α (Metallo *et al.*, 2012; Mucaj *et al.*, 2012; Mullen *et al.*, 2012), providing an alternative source of carbon to the TCA cycle which, during hypoxia, becomes largely disconnected from glycolysis (Daye & Wellen, 2012; Filipp *et al.*, 2012; Sun & Denko, 2014). Although this study does not enable clear definition of the metabolic pathways that utilize glutamine in VACV-infected cells, or of the role of HIF-1α in these metabolic changes, our observations are consistent with a model in which glutamine uptake and metabolism are upregulated to provide the biosynthetic precursors normally generated by glucose. However, due to the multi-functional role of C16, which can also block DNA-PK-mediated innate immune sensing by binding to the Ku complex (Ferguson *et al.*, 2012; Peters *et al.*, 2013), further studies are needed to understand whether other properties of this viral protein contribute to reprogramming of cellular metabolism.

Utilization of glutamine by VACV has been reported recently in two independent studies (Fontaine *et al.*, 2014; Greseth & Traktman, 2014). Consistent with our work, these studies highlighted the importance of glutamine as the main carbon source to preserve TCA cycle activity during VACV infection. Both studies also showed that whilst glutamine is required for viral protein synthesis, glucose starvation does not affect VACV replication. Although an increase in glycolysis was expected following HIF-1α stabilization and from the increase in glucose transporter 1 mRNA in cells infected with WT VACV compared with vΔC16 (Mazzon *et al.*, 2013), consistent with the results of Fontaine *et al.* (2014) and Greseth & Traktman (2014), our data did not show any increase in lactate production upon infection. However, increased concentration of the glucose-derived metabolites UDP-galactose and myo-inositol were observed (particularly in VACV-infected cells) and, although measurement of metabolite concentrations does not demonstrate unequivocally the pathways involved in their synthesis, it is possible that VACV uses glutamine to fuel the TCA cycle, and glucose is used as a carbon source for additional synthesis of nucleotides, phospholipids or macromolecules important for energy storage. Shunting of glucose towards biosynthetic pathways might explain in part why an increase in glycolysis and lactate production was not observed. A similar scenario has been described for human cytomegalovirus, which upregulates glucose uptake; however, glucose, rather than being completely broken down in the TCA cycle, is used biosynthetically for the synthesis of fatty acids. As a consequence, uptake of glutamine also increases and glutamine is converted into α-ketoglutarate to maintain the activity of the TCA cycle (Vastag *et al.*, 2011; Yu *et al.*, 2011).

In summary, this report shows that VACV infection increases utilization of glutamine using a different technique (^1^H-NMR) and two different cell lines. This study also demonstrates that while a number of viral proteins may influence the metabolic shift towards increased glutamine utilization, VACV C16 contributes to this shift and promotes the anabolic activity of infected cells. Mechanistically, protein C16 binds to PHD2 and thereby stabilizes HIF-1α during normoxia. However, HIF-1α stabilization does not promote aerobic glycolysis or the Warburg effect during VACV infection, but may instead contribute to the metabolic shift towards increased glutamine metabolism induced by C16.

## Methods

### 

#### Cell lines and viruses.

HeLa cells were cultured in MEM with 10 % FBS and 1 % (v/v) non-essential amino acids (Sigma). 2FTGH cells were cultured in Dulbecco’s modified Eagle’s medium (DMEM; Life Technologies) with 10 % FBS. VACV strain WR and vΔC16 lacking both copies of gene *C16L* were described previously (Fahy *et al.*, 2008). Viruses were grown in BSC-1 cells and purified from cytoplasmic extracts by sedimentation through a sucrose density cushion. Virus stocks were stored at −80 °C and the infectious titre was determined by plaque assay (Fahy *et al.*, 2008).

#### Immunoblotting.

Cells were lysed using a urea-based lysis buffer as described (Mazzon *et al.*, 2013). Primary antibodies used were: anti-C16 rabbit serum (Fahy *et al.*, 2008); anti-HIF-1α mouse monoclonal (BD Biosciences); anti-tubulin mouse monoclonal (Upstate Biotech). Bound antibodies were detected using fluorescent-conjugated goat anti-mouse or anti-rabbit secondary antibodies and infrared technology (Licor Biotechnology).

#### Quantitative PCR.

Cells were infected at 10 p.f.u. per cell and at the indicated times, DNA was extracted using a QiaAMP DNA mini kit (Qiagen). PCR analysis using specific primers for VACV gene *E9L* was performed with Fast SYBR Green Master Mix (Applied Biosystems) and analysed on a ViiA 7 instrument using ViiA 7 RUO software (Applied Biosystems). Primers used: DNA polymerase forward: 5′-ATGGATGTTCGGTGCATTAA; DNA polymerase reverse: 5′-GCATTAAATGGAGGAGGAGA.

#### Virus growth curves.

Confluent cells were infected at 10 p.f.u. per cell and harvested at 0, 12 and 24 h pi, and lysed by three freeze–thawing cycles. The infectious virus titre was quantified by plaque assay as described (Fahy *et al.*, 2008).

#### Infection and metabolite extraction.

Flasks of confluent HeLa or 2FTGH cells were infected at 10 p.f.u. per cell in MEM/1 % FBS/1 % (v/v) non-essential amino acids (Sigma) and cells were harvested at the indicated time points. Metabolites were extracted using a methanol/chloroform/water procedure. Six hundred microlitres of methanol/chloroform mix (2 : 1, v/v) were added to the cells and samples were sonicated for 15 min at room temperature. Two hundred microlitres each of chloroform and water were added, the samples were centrifuged (16 100 ***g***, 20 min) and the aqueous and lipid phases were collected. The procedure was repeated twice, and the aqueous and lipid fractions from each extraction were pooled. The aqueous layer was dried overnight in an evacuated centrifuge.

#### NMR analysis of aqueous extracts.

The dried extracts were rehydrated in 600 µl D_2_O water, containing 0.05 mM sodium-3-(tri-methylsylyl)-2,2,3,3-tetradeuteriopropionate (TSP; Cambridge Isotope Laboratories) as an internal standard. The samples were analysed using an AVANCE II+ NMR spectrometer operating at 500.13 MHz for the ^1^H frequency (Bruker) using a 5 mm TXI probe. Spectra were collected using a solvent suppression pulse sequence based on a one-dimensional nuclear Overhauser effect spectroscopy pulse sequence to saturate the residual ^1^H water signal (relaxation delay = 2 s, t1 increment = 3 us, mixing time = 150 ms, solvent pre-saturation applied during the relaxation time and the mixing time). One hundred and twenty-eight transients were collected into 16 K data points over a spectral width of 12 p.p.m. at 27 °C. In addition, generic cell samples were also examined by two-dimensional spectroscopy, including Correlation Spectroscopy, in conjunction with the Chenomix spectral database contained in Chenomix NMR Suite 7.0 (Chenomx) for spectral assignment.

#### NMR data processing.

NMR spectra were processed using an ACD one-dimensional NMR processor (version 12, ACD). Free induction decays were Fourier transformed following multiplication by a line broadening of 1 Hz, and referenced to TSP at 0.0 p.p.m. Spectra were phased and the baseline was corrected manually. Each spectrum was integrated using 0.02 p.p.m. integral regions between 0.5 and 4.5, and 5.5–8.5 p.p.m. The spectral region for each sample was scaled such that the total sum of integrals for each sample was equal. The integrals of the different metabolites were obtained using Chenomix.

#### Liquid chromatography MS analysis.

LC-MS analysis used a 4000 QTRAP triple quadrupole mass spectrometer (Applied Biosystems/Sciex), coupled to an Acquity UPLC (Waters) as described by Roberts *et al.* (2012), except that an Aquity UPLC (Waters) was used and multiple reaction monitoring parameters were added as outlined in Table S5. Formic acid, ammonium acetate, LC-MS-grade solvents and valine-d8 were from Sigma-Aldrich. Dried aqueous fractions from HeLa and 2FTGH cells were prepared for LC-MS analyses by the addition of 100 µl of 74.9 : 24.9 : 0.2 (by vol.) acetonitrile/methanol/formic acid containing the stable isotope-labelled internal standard valine-d8. Samples were vortexed, sonicated and the supernatants were injected directly.

#### Multivariate analysis of metabolite profiles.

The set of metabolic profiles obtained was analysed by multivariate analysis. Datasets were imported into SIMCA-P 12.0 (Umetrics) for processing using PCA and PLS-DA, a regression extension of PCA used for supervised classification. Briefly, in PLS-DA, the prior knowledge of class membership is available and a dummy Y matrix is created, which represents the class membership of each observation. A model is then fitted between the X and Y variables. The plane found is therefore discriminant because it maximizes the separation between classes. ^1^H-NMR data were Pareto scaled, in which each variable was centred and multiplied by 1/(Sk)1/2, where Sk is the standard deviation of the variable. Graphs were processed with GraphPad Prism 5.
